# A unifying theory of brain signaling and its possible role in acquired chronic disease

**DOI:** 10.3389/fnins.2026.1683413

**Published:** 2026-04-13

**Authors:** Gerard Pereira, Michael Corbett, Suzanne D. Vernon, Shaun Colburn, Sanjay Chanda

**Affiliations:** 1 Cortene Inc., Burlingame, CA, United States; 2 Bateman Horne Center, Salt Lake City, UT, United States; 3 Ballad Health CVA Heart Institute, Kingsport, TN, United States

**Keywords:** Alzheimer’s, chronic kidney disease, corticotropin-releasing factor (CRF), CRF receptor types 1 and 2 (CRFR1, CRFR2, CRF1, CRF2), dysregulation, Parkinson’s, serotonin

## Abstract

Acquired chronic disease is a significant, growing problem. Research has sought patient abnormalities that drugs can target, but to date, these have only provided equivocal symptom relief and no cures. However, as individual neuronal signals cannot be measured, the role of the brain in such diseases, has not been properly investigated. Here the authors propose that corticotropin-releasing factor (CRF) and serotonin act together in dedicated circuits, to architect precise, *bidirectional* signals that regulate normal function (e.g., thermoregulation, movement, memory, glomerular filtration rate). The authors propose further that the natural, circuit-specific upregulation of the CRF receptor type 2 (CRFR2), *unidirectionally* dysregulates these signals to cause chronic symptoms (e.g., low temperature, impaired movement, memory loss, reduced glomerular filtration rate). If confirmed, this view of chronic symptoms as a dysregulation of normal process via neuronal adaptation, has profound implications. It could explain Parkinson’s, Alzheimer’s and chronic kidney disease, among others, and, as it may be possible to downregulate CRFR2, could reverse the signs and symptoms of such diseases.

## 1 Introduction

*Acquired* chronic disease (ACD), developing in previously healthy individuals, accounts for 86% of years lived with disability, 71% of years of life lost (GBD 2019 Acute and Chronic Care Collaborators, 2025), and is projected to cost $47 trillion worldwide by 2030 (Hacker, 2024).

The prevailing approach has been to measure various parameters and identify abnormalities relative to healthy individuals. To date, such abnormalities have *not* been uniquely absent/present in patients, and only differ by degree from healthy persons. Without a clear understanding of how these parameters are governed, it is assumed that the abnormalities either cause or result from cellular damage, ultimately leading to chronic symptoms. This does not explain how such damage selects for the particular loci associated with symptoms, progresses slowly over decades, causes symptoms that fluctuate (good/bad days, flare with stress) or exhibit gender bias, yet can be absent in patients and present in healthy persons. It may explain why abnormality-targeting drugs are only modestly effective (Leucht et al., 2015).

One limitation of this approach is that it cannot easily measure individual neuronal signals (i.e., action potentials or neurotransmitter passage across synapses). Instead, it assesses receptors, transporters, synaptic/mitochondrial proteins, enzymes, metabolites, microglia, etc., often at singular timepoints (e.g., autopsy), from which broad neurotransmitter/neuromodulator signals are interpreted. Yet, broad levels of relatively few neurotransmitters/neuromodulators (7 major; ∼70 minor) could not support the range of human function or the need for ∼100 billion neurons. This suggests that signals need to be understood at the neuronal level. To date, there is no framework that delineates how these neuronal signals are individually architected, and continuously adapt for internal/external conditions, to regulate normal function.

The theory proposed here addresses these signals. It is based on an observed pattern across hundreds of independent studies, and supported by 2 clinical trials. This pattern has 3 elements: (i) corticotropin-releasing factor (CRF) and serotonin each modulate numerous individual functions (e.g., thermoregulation, movement, memory, glomerular filtration rate), and are further implicated in many ACDs; (ii) CRF and serotonin overlap throughout the brain; and (iii) CRF bidirectionally modulates serotonin release from the raphé nuclei, the source of serotonin in the brain and cord. This potentially implicates CRF and serotonin in the *normal* regulation of function, which could become *dysregulated* in ACD (Pereira et al., 2021). Here the authors propose a precise architecture by which neuronal signals could regulate normal function, and a specific mechanism by which the signals could become dysregulated to cause the symptoms of ACD.

## 2 Brain regulation of function

Determining how brain signals regulate function is simplified by 2 considerations. First, the brain is organized by function (Kandel et al., 2021), and independent functional operation necessitates *dedicated* circuits with defined origins and termini, but overlapping with, and receiving inputs from, numerous other circuits. Second, neurons *only* signal when the sum of their inhibitory and excitatory inputs exceeds a threshold (Kandel et al., 2021), so any change in the dedicated circuits must be triggered, and to enable functional prioritization across circuits, this likely involves a common trigger. CRF-serotonin could be such a trigger.

### 2.1 CRF triggers the circuits

The CRF system is present throughout the brain and is synonymous with the response to stress, where stress is anything that requires adaptive action (Selye, 1950; Selye, 1976). The CRF system consists of: 4 peptides, CRF and urocortin1, 2 and 3; CRF-binding protein; and 2 receptors, CRFR1 and CRFR2. CRF and urocortin1 bind to both receptors and CRF-binding protein; while urocortin2 and urocortin3 only bind to CRFR2 (Deussing and Chen, 2018; Dedic et al., 2018; Godoy et al., 2018). CRF is released under various conditions, including changes in homeostatic parameters (temperature, glucose, etc.), inputs from the autonomic, endocrine (Deussing and Chen, 2018; Dedic et al., 2018; Godoy et al., 2018) and immune (Nezi et al., 2000) systems, sensory cues (Liu et al., 2022; Graham et al., 2011; Shin et al., 2023; Heinrichs et al., 1991; Harrison and McLoon, 2006) including acute pain (Zheng et al., 2020), and even voluntary movement (Lowry et al., 1996; Wang et al., 2017). Thus, the CRF system could provide the impetus for initiating change in the functionally-dedicated circuits.

### 2.2 Brain serotonin marshals the circuits

Brain serotonin can act as a neurotransmitter via point-to-point synaptic contacts, or as a neuromodulator diffusing through volume transmission (Ligneul and Mainen, 2023). It is centrally synthesized in the raphé nuclei (Charnay and Léger, 2010) and projects to all parts of the brain and cord, with overlapping serotonin neurons individually synapsing onto multiple postsynaptic neurons, to enable broad influence over many functions (Aghajanian and Liu, 2009). It acts via multiple receptors (excitatory: 5HT_2–4_, _6–7_; inhibitory: 5HT_1_, _5_), of varying affinities, potencies (Harding et al., 2024) and electrophysiological effects (metabotropic: 5HT_1–2_, _4–7_; ionotropic: 5HT_3_) (Ligneul and Mainen, 2023; Charnay and Léger, 2010; Aghajanian and Liu, 2009; Berger et al., 2009). It modulates the major neurotransmitters, excitatory glutamate and inhibitory gamma-aminobutyric acid (GABA) (Ciranna, 2006; Celada et al., 2013), and the source nuclei of the major neuromodulators including dopamine (Esposito et al., 2008; Digiovanni et al., 2008; Courtiol et al., 2021), acetylcholine (Grace et al., 2012; Wu et al., 2021), histamine (Eriksson et al., 2001) and norepinephrine (Gorea and Adrien, 1988; Haddjeri et al., 1997), possibly via CRF (Snyder et al., 2012). It regulates the autonomic system (§2.7), the endocrine system via the hypothalamus (Dedic et al., 2018; Berger et al., 2009) and the immune system (Wan et al., 2020) via the vagus nerve (Jin et al., 2024; Tränkner et al., 2014). These characteristics uniquely qualify serotonin to marshal the dedicated circuits.

Yet, serotonin has been the focus of enduring misconceptions that oversimplify its role. Depression studies, showing low binding of the serotonin transporter (which recycles synaptic serotonin), were interpreted as *low* synaptic serotonin. This is potentially flawed because transporter expression decreases in the presence of serotonin (Ramamoorthy and Blakely, 1999; Gajeswski-Kurdziel et al., 2024), so low binding suggests *high* serotonin. This may explain why selective serotonin reuptake inhibitors, which block the transporter and increase synaptic serotonin immediately, only relieve depression 2–6 weeks later when compensatory action *decreases* serotonin (Machado-Vieira et al., 2010; Andrews et al., 2015; Moncrieff et al., 2022; Moncrieff et al., 2023). Before compensatory action, literally *any* side effect is possible (Ferguson, 2001; Warden et al., 2010; Andrews et al., 2012; Carvalho et al., 2016; Anagha et al., 2021; Edinoff et al., 2021), including alterations in neuromuscular control (Gönül and Aksu, 1999; Dixit, 2015; Uvais et al., 2016; Aamodt et al., 2022; Goldman and Galicia, 2022), cognition (Sayyah et al., 2016), interpreting social cues (Harmer et al., 2003), visual/auditory perception (Cancelli et al., 2004; Huot et al., 2012; Lai, 2012), intraocular pressure (Costagliola et al., 2008), taste, smell (Schiffman, 2018), autonomic reactivity (Agorastos et al., 2015), blood pressure (Calvi et al., 2021), lung function (Armstrong et al., 2022), esophageal motility (Manolakis et al., 2019), urinary incontinence (Movig et al., 2002), immune response (Snyder, 2011), etc. Similarly, life-threatening serotonin syndrome (i.e., drug-induced excess) causes neuromuscular symptoms (tremor, rigidity, hypertonicity, muscle spasms, etc.), autonomic hyperactivity, generalized seizures and mental state changes (Scotton et al., 2019). These data show that serotonin is involved in most bodily functions and interfering with it can impair function. Despite this, serotonin abnormalities invariably invoke depressive interpretations.

### 2.3 CRF-serotonin in the raphé

**FIGURE 1:**
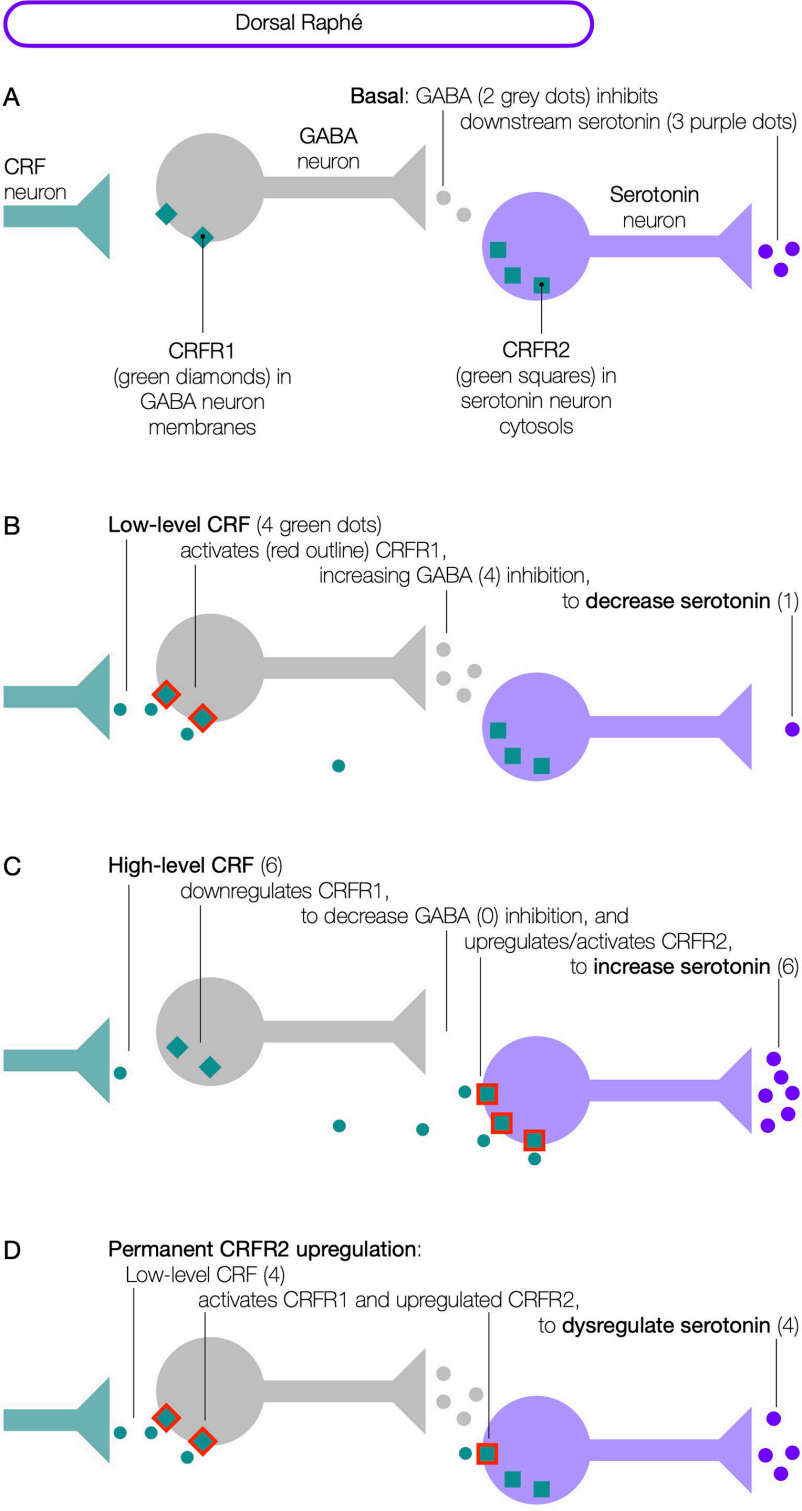
CRF modulates serotonin release from the dorsal raphé.

In rats, CRF modulates serotonin release from the dorsal raphé, the main source of brain serotonin (Waselus et al., 2009; Lukkes et al., 2008; Waselus et al., 2005). Within the raphé, CRFR1 is present in the membranes of GABA neurons, while CRFR2 is present in the cytoplasm of serotonin neurons (Figure 1A). This configuration, utilizing inhibitory GABA neurons, enables CRF to control serotonin release bidirectionally. That is, low-level CRF (Figure 1B), activates CRFR1 to release GABA, decreasing serotonin downstream; high-level CRF (Figure 1C), downregulates CRFR1, concomitantly upregulating and activating CRFR2 to increase serotonin downstream. Following such modulation, CRFR1 and CRFR2 internalize via receptor endocytosis (Markovic et al., 2008; Markovic et al., 2011; Hauger et al., 2013), and are then recycled to restore basal configurations (Deussing and Chen, 2018).

This property of escalating CRF activating first CRFR1 and then CRFR2 (via concomitant downregulation/upregulation of CRFR1/CRFR2), in conjunction with neuronal type, permits a high degree of control. That is, CRFR1 and CRFR2 expressed on the *same* neuron permits additive effect; while CRFR1 and CRFR2 expressed on *different* neurons can permit opposing effects (e.g., in the raphé) or complementary effects.

### 2.4 CRF-serotonin in the brain

**FIGURE 2:**
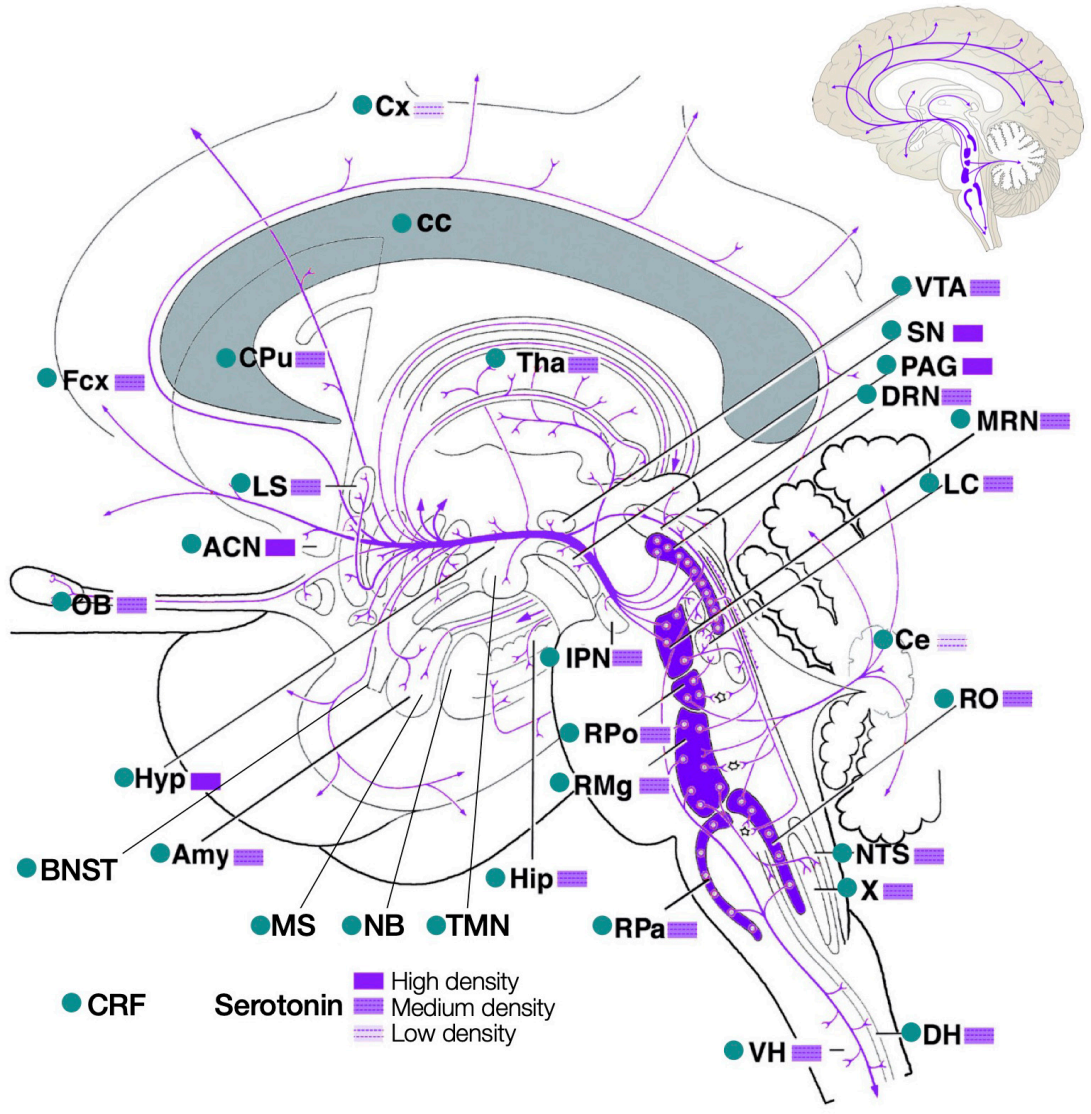
CRF (green dots) and serotonin nuclei (purple) interact throughout the brain, including the source nuclei of dopamine (SNpc, VTA), acetylcholine (IPN, MS, NB), histamine (TMN) and norepinephrine (LC). ACN, accumbens nucleus; Amy, amygdala; BNST, bed nucleus of stria terminalis; cc, corpus callosum; Ce, cerebellum; CPu, caudate-putamen; Cx, cortex; DH, dorsal horn spinal cord; DRN, dorsal raphé nucleus; Fcx, frontal cortex; Hip, hippocampus; Hyp, hypothalamus; IPN, interpeduncular and pedunculopontine nuclei; LC, locus coeruleus; LS, lateral septum; MRN, median raphé nucleus; MS, medial septal nuclei; NB, nucleus basalis; NTS, nucleus of the solitary tract; OB, olfactory bulb; PAG, periaqueductal gray; RMg, raphé magnus nucleus; RO, raphé obscurus nucleus; RPa, raphé pallidus; RPo, raphé pontis nucleus; SN, substantia nigra, both SNpc (pars compacta) and SNpr (pars reticulata); Tha, thalamus; TMN, tuberomammillary nucleus; VH, ventral horn; VTA, ventral tegmental area; X, dorsal motor nucleus of vagus nerve. Adapted from Deussing and Chen, 2018; Dedic et al., 2018; Godoy et al., 2018; Charnay and Léger, 2010.

**FIGURE 3:**
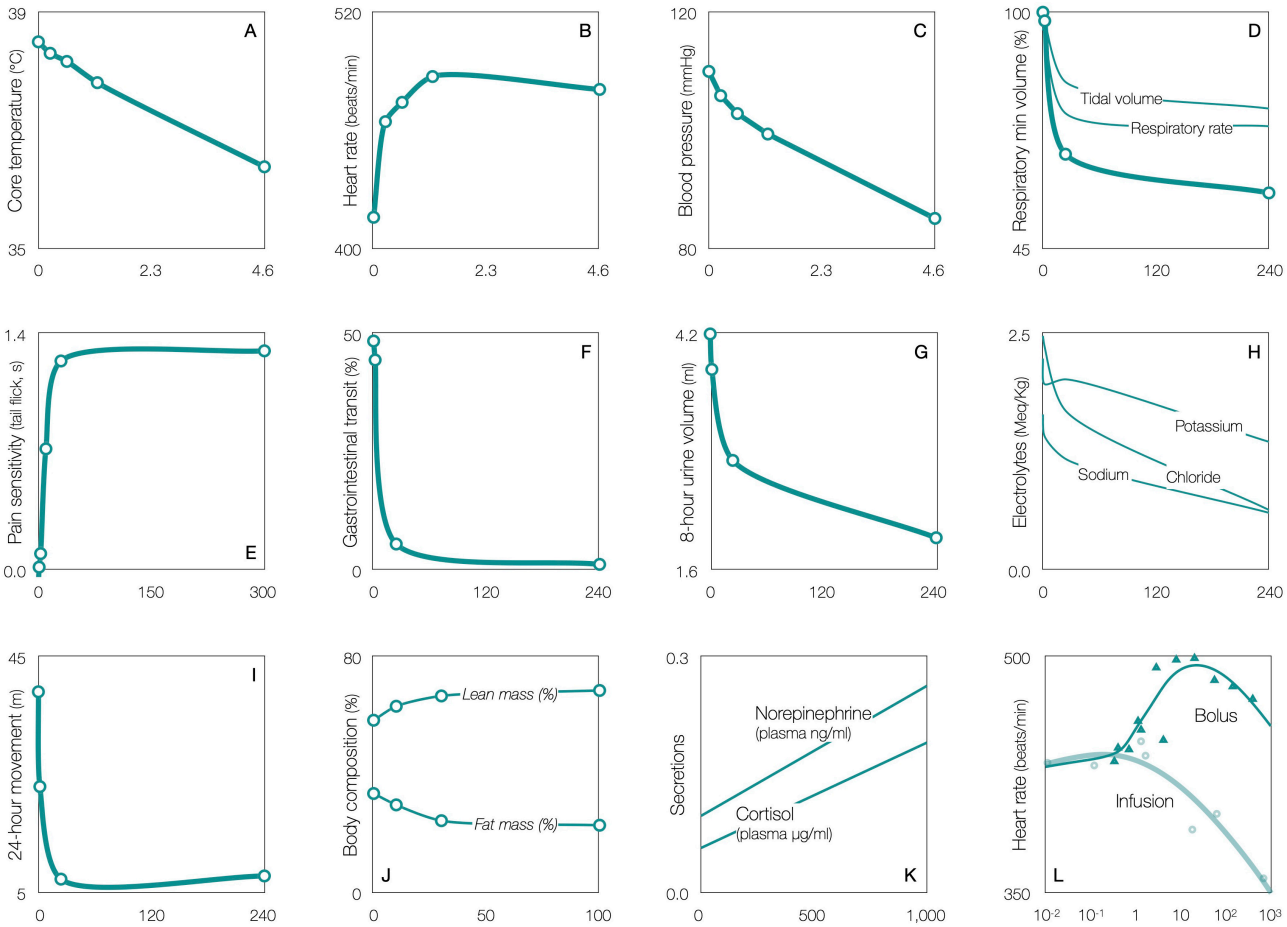
CRFR2 stimulation dose-dependently regulates numerous functions in rats. Effect of escalating doses (μg/Kg, x-axis) of CT38 (a short-lived peptide agonist that binds only to CRFR2) on various functions (y-axis). Adapted from Pereira et al., 2021.

The CRF and serotonin systems overlap throughout the brain (Figure 2; Deussing and Chen, 2018; Dedic et al., 2018; Godoy et al., 2018; Charnay and Léger, 2010). Given this overlap, and based on their known interaction in the raphé, the authors propose that CRF also triggers the dedicated circuits, activating local CRFR1/CRFR2, to modulate serotonin and regulate individual bodily functions. For instance in rats, CT38, a short-lived peptide agonist that binds *only* to CRFR2, dose-dependently affects temperature, heart rate, blood pressure, breathing (rate and volume), pain sensitivity, gastrointestinal transit, urine production (and electrolytes), movement, body composition (lean/fat mass) and the release of norepinephrine and corticosterone (Figure 3; Pereira et al., 2021).

### 2.5 Architecting the net serotonin effect

**FIGURE 4:**
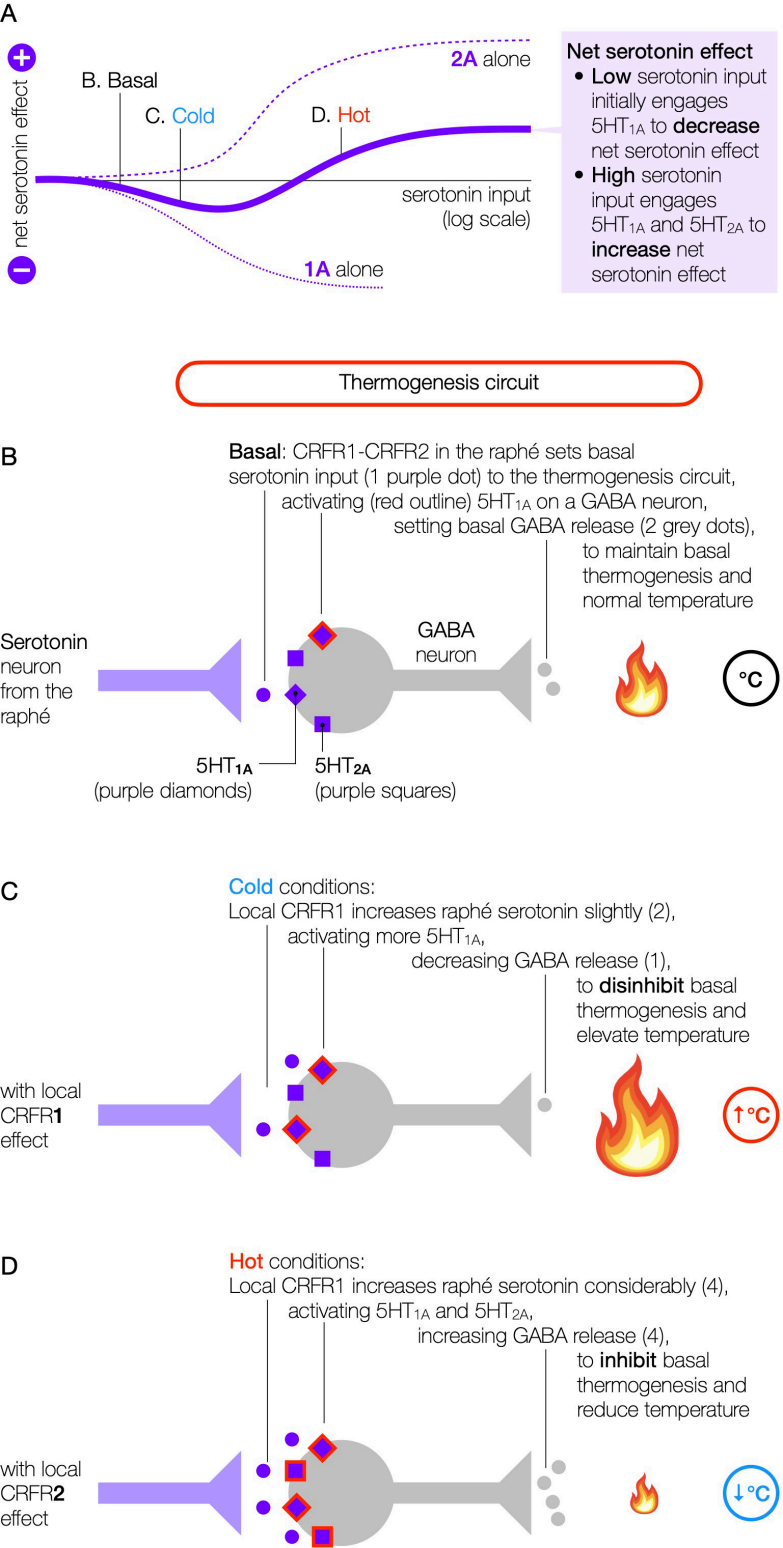
Architecting circuit signals.

The foregoing provides a plausible basis for suggesting the means by which CRF-serotonin architects circuit-specific trigger signals in the dedicated circuits. For an individual circuit, serotonin input from the raphé, further modulated by *local* CRF, activates both inhibitory (e.g., 5HT_1A_) and excitatory (e.g., 5HT_2A_) receptors on *effector* neurons (Celada et al., 2013; Lambe et al., 2011). The resulting *net serotonin effect* at the axon hillock (Kandel et al., 2021; Ligneul and Mainen, 2023) of the effector neurons (Figure 4A) is influenced by receptor affinity (e.g., 5HT_1A_ > 5HT_2A_), receptor density and intracellular proteins (Gi or Gq), which attenuate 5HT_1A_ (Raymond et al., 1999) or amplify 5HT_2A_ (Wallach et al., 2023) effects. Downstream action then depends on this net serotonin trigger signal and the type of effector neuron.

By way of example, thermoregulation is known to involve a circuit projecting from the dorsal raphé to the preoptic-anterior hypothalamus that controls thermogenesis via inhibitory GABA effector neurons. The authors propose that the circuit-specific CRFR1-CRFR2 configuration in the raphé, determines basal serotonin, in turn setting basal GABA release and basal thermogenesis to maintain normal body temperature (Figure 4B). Changes in body temperature release a transient (phasic) CRF trigger that locally modifies basal serotonin to architect a *situationally-specific, net serotonin effect*. Thus in cold conditions, *low-level* CRF activates local CRFR1 on the serotonin neuron from the raphé, increasing basal serotonin slightly, which activates 5HT_1A_ to decrease GABA release, thereby disinhibiting basal thermogenesis and elevating temperature (Figure 4C). In hot conditions, *high-level* CRF downregulates local CRFR1 and upregulates/activates local CRFR2 (on the serotonin neuron from the raphé) to increase basal serotonin considerably, which activates *both* 5HT_1A_ and 5HT_2A_ to increase GABA release, thereby inhibiting basal thermogenesis and reducing temperature (Figure 4D). Note that other serotonin receptors (e.g., 5HT_1B_, 5HT_2C_), with different affinities, potencies and densities, might also be expressed (Kandel et al., 2021; Ligneul and Mainen, 2023), allowing for more finely tuned signals. Similar changes in parallel circuits mediate complementary effects on vasoconstriction, shivering, piloerection, vasodilation, sweating, etc., resulting in a coordinated thermoregulatory response. Note further that temperature changes are not just reactive, but can be proactive as well, e.g., diurnal variation (Miller et al., 2024), response to infection, etc.

This construct is supported by rat studies showing that hypothalamically activating CRFR1 (Figueiredo et al., 2010) or GABA_A_ receptors (which decreases GABA) (Ishiwata et al., 2005), elevates temperature; while activating CRFR2 (Figure 3A) or blocking GABA_A_ (which increases GABA) (Ishiwata et al., 2005), reduces temperature. Yet, selectively activating 5HT_1A_ or 5HT_2_, hypothalamically in rats (Lin et al., 1998) or orally in humans (Anderson et al., 1990; Shick Lee et al., 1992), *ostensibly* decreasing and increasing serotonin, respectively reduces and elevates temperature—apparently reversed. This highlights another potential flaw in serotonin research. The use of an *unnatural* receptor-selective agent for 5HT_1A_ or 5HT_2_, can confirm the presence of the target receptors in the circuit, but it corrupts the net serotonin effect by increasing the binding of *natural* serotonin to the opposite receptor, further confounded by dose, binding affinities, receptor densities and relative Gi/Gq activity. Thus, this paper cites studies that demonstrate the presence of inhibitory/excitatory receptors, but does not utilize their inferences.

Thermogenesis is *on* by default, which GABA effector neurons can disinhibit/inhibit. In other functions such as chronic pain (*not* on by default), glutamate effector neurons might be more appropriate (Pereira and Goudet, 2019). Serotonin modulates both glutamate and GABA extensively throughout the brain (Ciranna, 2006), with its circuit-specific effect being either inhibitory or excitatory depending upon effector neurons. Complex functions such as movement, likely involve multiple coordinated circuits that utilize the situationally-specific net serotonin trigger signals to elicit other neuromodulator input, e.g., dopamine (Alex and Pehek, 2007), acetylcholine (Nair and Gudelsky, 2004) and norepinephrine (Done and Sharp, 1994), respectively associated with value determination (Berke, 2018), neuronal excitability (Picciotto et al., 2012) and arousal (Maness et al., 2022). Such input sculpts the downstream serotonin signals to regulate function precisely.

This proposed pattern of CRF modulating serotonin, in turn activating inhibitory and excitatory receptors to architect bidirectional, situationally-specific, net serotonin signals, is widely evident in regulating normal function (§3).

### 2.6 Permanent CRFR2 upregulation dysregulates the circuit signals

In rats, under intense or repetitive stress, CRFR2 can fail to downregulate in the raphé, and remain permanently upregulated (Lukkes et al., 2009; Wood et al., 2013). Then, low-level CRF that would have activated only CRFR1 to decrease serotonin, instead activates both CRFR1 and upregulated CRFR2 to increase serotonin (Figure 1D). The authors propose that permanent CRFR2 upregulation also occurs in the dedicated circuits, where instead of low-level CRF activating CRFR1 to increase serotonin slightly, it activates both CRFR1 and upregulated CRFR2 to increase serotonin considerably, thereby increasing the net serotonin effect (Figure 4A) and disrupting downstream function to cause a chronic symptom. For instance, based on rats, CRFR2 upregulation in thermoregulatory or respiratory circuits would be expected to inhibit thermogenesis or diaphragmatic contraction, lowering body temperature or breathing (Figures 3A, D)—both of which are evident in aging (Waalen and Buxbaum, 2011; Van Mourik et al., 2014). This suggests that CRFR2 upregulation in a given circuit, can both raise basal serotonin and/or bias the sensitivity of the response to provocation.

While replicable in animals, the exact mechanism by which CRFR2 fails to downregulate is not known. It appears to involve excess serotonin (Rozeske et al., 2011). Acute stress releases CRF immediately, which dissipates rapidly. However, intense/repetitive stress releases urocortin1, which peaks at 2–4 hours and remains active for 18 hours (Kozicz, 2007), likely maintaining elevated serotonin over a period long enough to prevent CRFR2 downregulation. This urocortin1-serotonin mechanism may be adaptive, intended to upregulate CRFR2 and induce resilience to particular stimuli (Wood et al., 2013; Wood, 2014; Wood and Bhatnagar, 2015; McEwen, 2017; Faye et al., 2018; Bhatnagar, 2021; Chaves et al., 2021, Kageyama et al., 2021; Pomrenze et al., 2021), but it may have other consequences.

### 2.7 Implications of CRFR2 upregulation

The foregoing describes CRFR1 and CRFR2 *adapting* to modulate serotonin, in turn architecting a net serotonin effect that marshals other neurotransmitters/neuromodulators to regulate normal function. Under certain conditions, this natural adaptation can become permanent, via CRFR2 upregulation, thereby elevating serotonin to bias the circuit, generally more towards inhibition than excitation. If this proposed pathogenesis of ACD is confirmed, it could have important implications, including:

- *Chronic symptoms represent dysregulated function.* This arises when excess serotonin, in a circuit being modulated, induces permanent CRFR2 regulation, thereby biasing that circuit’s normal control of function in a particular direction. For instance, narrow, repeat provocations, such as risky pursuits in rats (Freels et al., 2020) and novel (Zald et al., 2008) or creative (Zabelina et al., 2016) pursuits in humans, engage dopamine circuits in gender-specific ways (Byrnes et al., 1999), which may upregulate CRFR2. This would inhibit dopamine release, potentially affecting other dopamine-utilizing circuits (e.g., movement), explaining why these behaviors are risk factors for Parkinson’s (Voon et al., 2011; Clark and Dagher, 2014; Grover et al., 2019). Intense provocations, e.g., COVID-19, may upregulate CRFR2 across many circuits to cause numerous symptoms (Davis et al., 2021). Milder provocations, e.g., early life stress, may also upregulate CRFR2 widely, though insufficiently to cause overt symptoms, but cumulative stress may reach symptom threshold. This may explain why chronic symptoms overlap across ACD (Table 1), depend on personal history (prior CRFR2 upregulations), flare with stress (releases CRF), progress (circuits operating at increased serotonin are prone to serotonin excess and further CRFR2 upregulation), and persist (unless CRFR2 downregulates).
- *Provocations* like microbes, toxins, physical/mental trauma, etc. (Pereira et al., 2021), all release CRF so may induce CRFR2 upregulation. This partially disconnects the provocation from its chronic symptoms, i.e., a virus might induce CRFR2 upregulation, but need *not* be present for symptoms to persist. This may explain how microbes like COVID-19 (Jackson et al., 2022) and *Borrelia burgdorferi* (Petnicki-Ocwieja and Kern, 2014) can differ in cellular targets and entry mechanisms, yet exhibit similar chronic symptoms in the absence of the microbe, in long COVID and chronic Lyme disease, respectively.
- *Individual ACDs* possibly develop based on the proximity of the circuits involved in their core symptoms, as CRF, urocortin1 and serotonin can act broadly within a region (i.e., volume transmission), further influenced by circuit activity/inactivity (§2.7, “Neurons are dynamic”) and certain genes. Note however, that ACDs are *not* distinct entities, as their chronic symptoms are not ACD-specific (Table 1); they are related as having an ACD increases the likelihood of additional ACDs (Buttorff et al., 2017); and their incidences increase with life/cumulative stress in gender-specific ways, evident in both the CRF (Bangasser et al., 2010, 2013; Bangasser, 2013; Howerton et al., 2014; Weathington et al., 2014; Lukkes et al., 2016) and serotonin (Philippe et al., 2022) systems.
- *Autonomic dysfunction.* The autonomic system is centered on the hypothalamus and certain nuclei in the brain stem, e.g., nucleus of the solitary tract, periaqueductal gray, parabrachial nucleus (Saper and Stornetta, 2015; Gibbons, 2019). It affects temperature, heart rate, blood pressure/flow, breathing rate/volume, digestion, urine production/electrolytes, fluid volume, glandular secretions, glucose homeostasis, immune function, sexual function, etc. (Wehrwein et al., 2016). The brain stem is involved in stress response, so frequent/excessive stress could conceivably upregulate CRFR2 to cause autonomic dysfunction (Figure 3), which is widely evident across ACD (Table 1).
- *Serotonin-marshaled circuits* involves serotonin in many individual functions, i.e., serotonin is just as involved in depression as it is in thermoregulation or breathing. It also implies that serotonin dysregulation in a given circuit would likely disrupt other neurotransmitters/neuromodulators in the circuit.
- *Precision and specificity* are paramount. In Sprague-Dawley rats, intracerebroventricular CRF doses of 0.025 and 0.1 μg have *opposite* effects (Hupalo and Berridge, 2016; Hupalo et al., 2019, 2021), and Figure 3 shows wide dose- and duration-dependent CRFR2 effects, together implying that function is controlled by precise signals in specific circuits. Serotonin is equally precise, e.g., transporter expression varies with synaptic serotonin *in vitro* (Ramamoorthy and Blakely, 1999; Gajeswski-Kurdziel et al., 2024), and by individual, brain region (Rylands et al., 2012) and even season (Praschak-Rieder et al., 2008) in humans. This necessitates cautious interpretation of experimental studies.
- *Neurons are dynamic* and neuronal activity induces remodeling (Chen and Nedivi, 2010; Lavoie-Cardinal et al., 2020; Pan and Monje, 2020; Wiesner et al., 2020; Mayseless et al., 2023), with short- and long-term changes in receptors (Citri and Malenka, 2008), transporters (Ramamoorthy and Blakely, 1999; Gajeswski-Kurdziel et al., 2024), synapses (Fisher-Lavie and Ziv, 2013; Okabe, 2007), mitochondria (Devine and Kittler, 2018; Duarte et al., 2023) and microglia (Pallarés-Moratalla and Bergers, 2024). For instance, 14-day right arm immobilization in right-handed humans, so reducing sensorimotor neuronal signaling, *decreases* cortical thickness in the *left* sensorimotor cortex and corticospinal tract (which control the right arm), but *increases* thickness on the *right*, correlating with improvements in left-arm motor skills (Langer et al., 2012)—showing that even short-term signaling changes can alter neuronal structure. Similarly, normal aging involves minor reductions in neuronal count and major losses in cortical mass and dendritic architecture (Terry et al., 1987; Freeman et al., 2008; Von Bartheld, 2018). Thus, apparent abnormalities in neurotransmitters/neuromodulators, receptors, transporters, synapses, enzymes, mitochondria, microglia, etc., might only reflect circuit-specific activity, and *not* neurodegeneration, which requires evidence of reduced neuronal counts at autopsy. Permanent CRFR2-induced inhibition may thus cause neuronal atrophy, which may eventually lead to pre-mortem neuronal death from loss of signal (Fricker et al., 2018).
- *Protein aggregates.* Alpha-synuclein protein, implicated in Parkinson’s (Siderowf et al., 2023), is involved in serotonin transporter activity (Wersinger et al., 2006) and synaptic cargo release (Sulzer and Edwards, 2019); while amyloid precursor and tau proteins, implicated in Alzheimer’s (Knopman et al., 2021), are respectively involved in synaptic function (Tyan et al., 2012) and axonal transport, synaptic structure and signaling (Tapia-Rojas et al., 2019). These *signaling* proteins cannot be synthesized in realtime, so are pre-synthesized. Under CRFR2-induced neuronal inhibition, such proteins would remain unused so could aggregate. This might explain the existence of such aggregates across ACDs, e.g., alpha-synuclein aggregates occur in Parkinson’s, multiple system atrophy (Singer et al., 2020), Alzheimer’s (Korff et al., 2013), Creutzfeldt-Jakob disease (Kruse et al., 2018), Huntington’s (Breza et al., 2020), amyotrophic lateral sclerosis (Smith et al., 2024) and multiple sclerosis (Wang et al., 2012); amyloid beta brain plaques occur in Alzheimer’s, type 2 diabetes and hypertension without dementia (Van Arendonk et al., 2023); while tau neurofibrillary tangles occur in Alzheimer’s, Pick’s disease, progressive supranuclear palsy, corticobasal degeneration, and argyrophilic grain disease (Medeiros et al., 2011).

**TABLE 1:** Shared symptos of ACD.

	PD	AD	Dys	ME/CFS	FM	CKD	MS	RA	T2D	HT	CRFR2
**Brain fog** (cognition, memory, attention)	●	●	●	●	●	●	●	●	●	●	**✓**
**Movement** (slowness, incoordination, involuntary, tremor)	●	●	○	●	●	●	●	●	○	○	**✓**
**Fatigue** (weakness, exercise intolerance)	●	●	●	●	●	●	●	●	●	●	**✓**
**Sensory** (sight, hearing, smell, taste, touch, pain)	●	○	●	●	●	●	●	●	○	●	**✓**
**Sleep** (insomnia, disturbance, restlessness)	●	●	○	●	●	●	●	●	●	●	**✓**
**Mood** (anxiety, apathy, anger, depression, swings)	●	●	●	●	●	○	○	○	●	●	**✓**
**Flaring under stress**	●	●	●	●	●	○	●	●	●	●	**✓**
**Temperature** (fever, chills)	●	○	●	●	●	○	●	●	○	●	**✓**
**Cardiovascular** (heart rate, hypotension, orthostatic intolerance)	●	○	●	●	●	●	●	●	○	●	**✓**
**Shortness of breath**	○	○	●	●	●	●	●	●	●	●	**✓**
**Flu-like symptoms**	○	●	○	●	●	○	○	●	●	●	**✓**
**Digestion** (swallowing, nausea, appetite leaky gut, IBS)	●	○	●	●	●	●	●	○	○	○	**✓**
**Urination** (incontinence, overactivity, urine chemistry)	●	○	●	●	●	●	●	●	○	○	**✓**
**Immune dysfunction**	●	○	○	●	●	○	●	●	●	●	**✓**
**Insulin resistance** (T2D)	○	●	●	●	●	●	●	●	●	●	**✓**
**Hypothyroidism** (HT)	○	○	○	●	●	●	○	○	○	●	**✓**
**Serotonin involvement**	**✓**	**✓**	**✓**	**✓**	**✓**	**✓**	**✓**	**✓**	**✓**	**✓**	

PD, Parkinson’s disease; AD, Alzheimer’s disease; Dys, dysautonomia; ME/CFS, myalgic encephalomyelitis/chronic fatigue syndrome; FM, fibromyalgia; CKD, chronic kidney disease; MS, multiple sclerosis; RA, rheumatoid arthritis; T2D, type 2 diabetes; HT, hypothyroidism; IBS, irritable bowel syndrome. ● core symptom; ○ non-core symptom; **✓** evidence that CRFR2 and serotonin are implicated in these symptoms and diseases.

### 2.8 Downregulating CRFR2

The authors conducted a 14-patient proof-of-concept trial, seeking evidence that CRFR2 might be upregulated in myalgic encephalomyelitis/chronic fatigue syndrome (a multi-symptom ACD of unknown cause), which a limited infusion of CT38 might downregulate, resulting in permanent symptom reduction (Pereira et al., 2021)—summarized here for convenience. All patients received CT38 at 1 of 4 *blinded* concentrations for a set period of time. While this small trial needs validation, patient effects correlated with *objective* blood levels of CT38.

**FIGURE 5:**
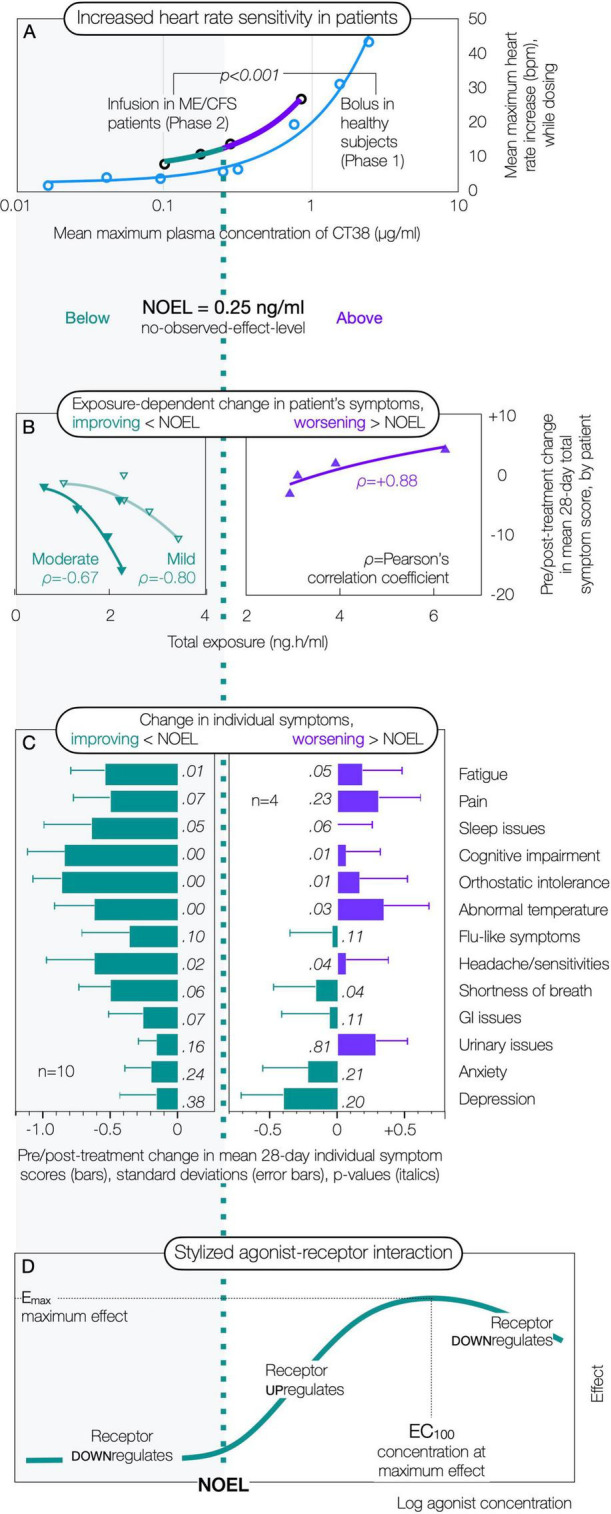
Effect of infused CT38 at different concentrations in myalgic encephalomyelitis/chronic fatigue syndrome (ME/CFS). Adapted from Pereira et al., 2021.

- Patients showed greater heart rate sensitivity to CT38 concentrations than healthy subjects in prior Phase 1 trial (Figure 5A). This is consistent with CRFR2 upregulation, especially given the relative effects of CT38 by bolus or infusion in rats (Figure 3L), suggesting normal regulation of function (heart rate) becoming dysregulated with increased sensitivity in patients.
- Patients showed the same *no-observed-effect-level* (i.e., the concentration below which there is no observable effect, and above which effect increases substantially as receptors upregulate) as healthy subjects, but patients had a higher basal effect (Figure 5A), both consistent with CRFR2 upregulation.
- Subjectively-assessed change in total symptom score by patient, increased with total exposure, *worsening above* the no-observed-effect-level and *improving below* (Figure 5B). Individual symptom scores showed this same pattern (Figure 5C). These findings are consistent with CRFR2 upregulation and downregulation—the latter further supported by the duration of effect, which was sustained over months (to years, via informal follow-up). Importantly, receptor downregulation can only be demonstrated by reduced effect, so requires high concentrations *in vitro* (Markovic et al., 2008; Markovic et al., 2011; Hauger et al., 2013) or *in vivo* (Figure 3L). The *inferred* downregulation at low concentrations in the trial is consistent with recent *in vitro* work demonstrating that it is possible (Pack et al., 2018), and the infusion duration required to downregulate CRFR2 (∼20 hours by extrapolation of Figure 5B, regardless of symptom severity) matches the extended release of urocortin1 (18 hours) that may induce upregulation (Kozicz, 2007). A therapeutic approach using concentrations below the no-observed-effect-level (Figure 5D) would not be expected to upregulate the receptor, so if it induces downregulation, this might be *selective* for only those neurons where the receptor is upregulated.
- The foregoing suggests that the CRF dose-response is complex. Bolus CRF concentrations above the no-observed-effect-level, activate CRFR1 (Figure 5D); higher concentrations activate CRFR2 while downregulating CRFR1 (Waselus et al., 2009); still higher concentrations, beyond maximum effect, downregulate CRFR2 (Figure 3L). Infused CRF appears to downregulate CRFR2 at concentrations much lower than at maximum effect by bolus (Figure 3L). Other CRF-related peptides probably have similar profiles, but at different threshold concentrations. These effects necessitate cautious experimental interpretation.

## 3 CRF-serotonin in ACD

The foregoing proposes that CRFR2 upregulation causes chronic symptoms and ACD. The following sections present data supporting the connection between the CRF-serotonin system and Parkinson’s disease, Alzheimer’s disease and chronic kidney disease, and more broadly in systemic regulation.

### 3.1 Parkinson’s disease

Parkinson’s progresses over decades, from non-motor symptoms such as loss of smell, impaired cognition, disturbed sleep, autonomic dysfunction (Santiago et al., 2017), to motor symptoms such as rest tremor, muscle rigidity, slowed movement, flexed posture, freezing of gait and postural instability (Obeso et al., 2017), all of which flare with stress (van der Heide et al., 2021). Patient scans show abnormalities in serotonin (Politis and Niccolini, 2015), glutamate (Carrillo-Mora et al., 2013), GABA (Błaszczyk, 2016), dopamine (Obeso et al., 2017), acetylcholine (Bohnen et al., 2019), histamine (Rinne et al., 2002), norepinephrine (Paredes-Rodriguez et al., 2020), mitochondrial proteins (Henchcliffe and Beal, 2008), synaptic vesicle proteins (Holmes et al., 2024), and activated microglia (Stefanova, 2022). Known risks include early (Dallé and Mabandla, 2018) and cumulative (Vlajinac et al., 2013) life stress, antidepressant usage (Alonso et al., 2009), risky (Voon et al., 2011), novel (Clark and Dagher, 2014) or creative (Grover et al., 2019) pursuits, gender (Haaxma et al., 2007), and certain genes (Day and Mullin, 2021; Tran et al., 2020). Autopsies suggest reduced counts of dopamine neurons projecting from the substantia nigra pars compacta (nigra) to the dorsal striatum (Fearnley and Lees, 1991), with deficits in tyrosine hydroxylase (Heo et al., 2020), the enzyme involved in synthesizing dopamine and neuromelanin (Nagatsu et al., 2023). Autopsies also show intracellular Lewy bodies (protein aggregates containing alpha-synuclein and other cellular proteins) (Simon et al., 2020; Iwatsubo et al., 1996; Pollanen et al., 1993) in the brain stem, but not consistently in the nigra (Obeso et al., 2017).

Prevailing ideas center on alpha-synuclein misfolding, inducing more misfolding, accumulating as Lewy bodies, which spread in a prion-like manner to destroy neurons (Obeso et al., 2017). Yet, there is no *direct* evidence of misfolding or prion-like spread (Walsh and Selkoe, 2016), and Lewy bodies can be present in healthy persons (Markesbery et al., 2009), absent in patients (Parkkinen et al., 2011; Johansen et al., 2018), do not destroy neurons (Tompkins and Hill, 1997), follow not precede symptoms (Milber et al., 2012), and are not specific to Parkinson’s (Singer et al., 2020; Korff et al., 2013; Kruse et al., 2018; Breza et al., 2020; Smith et al., 2024; Wang et al., 2012; Halliday et al., 2011). Not surprisingly, a systematic review of the literature shows that alpha-synuclein/Lewy bodies, fail the minimum Bradford Hill causation criteria in Parkinson’s (Espay et al., 2019), suggesting that Lewy bodies maybe more of an effect than the cause.

The following studies support the case for CRFR2 upregulation in motor and autonomic circuits, leading to serotonin dysregulation and symptoms in Parkinson’s.

- *Nigral inhibition.* In rats, optogenetically inhibiting nigral dopamine over 24 hours, induces severe motor and tyrosine hydroxylase deficits lasting 5 days, but recovering completely (Heo et al., 2020). In Parkinson’s models (A53T and 6-hydroxydopamine), optogenetically activating nigral dopamine neurons rescues motor and tyrosine hydroxylase deficits, suggesting that these models do *not* destroy neurons (Heo et al., 2020). This study shows that solely inhibiting nigral dopamine is sufficient to cause certain Parkinson’s signs and symptoms, which disinhibition rescues.
- *CRF induces certain signs and symptoms.* In rodents (Muramatsu et al., 2006; Kim et al., 2009; Steger et al., 2020; Mor et al., 2022), monkeys (Kelly et al., 2024) and humans (Payer et al., 2017), acutely activating CRFR1 releases dopamine, while acutely activating CRFR2 (or following repetitive stress, which upregulates CRFR2) suppresses dopamine—and inhibitory/excitatory serotonin receptors bidirectionally modulate dopamine (Alex and Pehek, 2007). In rats, at CRFR2-activating doses (Hupalo and Berridge, 2016; Hupalo et al., 2019, 2021), acute CRF administration induces tremor (Jones et al., 1998), while repetitive CRF administration (intracerebroventricular injections for 13 days) causes muscle rigidity and reduces movement (Izzo et al., 2005). Downregulating cerebellar CRF (by lentivirus) impairs gait (shorter stride length), coordination (less time on a rota-rod) and balance (longer time to cross a beam), likely by *not* releasing dopamine—all rescued by CRF microinjection (Wang et al., 2017). This connects the dopamine signs and motor symptoms of Parkinson’s with the CRF system, in a manner consistent with CRFR2 upregulation.
- *CRF is effective in rat models* (Abuirmeileh et al., 2007a,b, 2008, 2009), where a toxin (lipopolysaccaride or 6-hydroxydopamine) time-dependently suppresses nigral/striatal dopamine and impairs motor function. Acutely activating CRFR1, but not CRFR2, rescues function, consistent with CRFR1/CRFR2 releasing/suppressing dopamine. Such rescue occurs even *after* the toxin has supposedly destroyed ∼80–90% of the neurons, again showing that these models do *not* destroy neurons. This emphasizes the involvement of CRFR1 in releasing dopamine, which CRFR2 upregulation would suppress.
- *Autopsies* show 60–70% decreases in CRF and choline acetyltransferase (involved in acetylcholine synthesis) in the frontal, temporal and occipital cortices (Whitehouse et al., 1987), with related work showing reciprocal increases in the CRF receptors (De Souza et al., 1986). This supports the involvement of the CRF system in Parkinson’s.

Circuit-specific CRFR2 upregulation and serotonin dysregulation may explain several observed abnormalities, including: dopamine suppression, with tyrosine hydroxylase deficit (Heo et al., 2020); muscle rigidity (Izzo et al., 2005); impaired gait, coordination and balance (Wang et al., 2017); tremor (Jones et al., 1998), which correlates better with serotonin (Doder et al., 2003) than dopamine (Obeso et al., 2017); rest tremor in patients whose scans are without evidence of dopamine deficit, so-called SWEDD (Obeso et al., 2017; Erro et al., 2016; Sasaki, 2024); and similarity with the neuromuscular symptoms of excess serotonin (Scotton et al., 2019). It may explain non-motor symptoms, including: autonomic (§2.7); sleep disorders, which are modulated by CRF (Ono et al., 2020) and serotonin (Berger et al., 2009); mood changes, which are related to serotonin (Berger et al., 2009) and not dopamine (Radad et al., 2023); and loss of smell, which is dopamine-related (Rampin et al., 2022), but enriched by CRFR1 (Garcia et al., 2016). It may explain slow progression, stress-induced symptom-flaring, and the influence of early life/cumulative stress, antidepressant usage, gender, and risky pursuits (§2.7). It may explain Parkinson’s-associated genes, which elevate intracellular calcium (Zaichick et al., 2017), though insufficiently for atrophy and adolescent onset, but in conjunction with CRFR2 upregulation, which also elevates intracellular calcium (Deussing and Chen, 2018), might be sufficient to induce atrophy.

Serotonin dysregulation may explain changes in other neurotransmitters/neuromodulators (§2.7), including: dopamine suppression (Alex and Pehek, 2007); declining levodopa effect (Tanner, 2020) under serotonin-increasing situations, such as stress, anxiety/depression or fatigue (Mantri et al., 2021) then leading to serotonin-related dyskinesia (Carta and Tronci, 2014); acetylcholine, leading to falls, freezing of gait (Bohnen et al., 2019) and dementia (Hall et al., 2014); and paradoxical kinesia (Distler et al., 2016), as new circuit inputs (sensory cues, cognitive/emotional stimuli, danger, etc.) cause the dopamine system to assign a higher value to the intended action (Berke, 2018), releasing nigral dopamine and normalizing movement. It may explain neuronal atrophy resulting from chronic inhibition (§2.7), centered on the nigra and progressing along network connectivity pathways (Tremblay et al., 2021; Giguère et al., 2018; Yau et al., 2018; Zeighami et al., 2015). Finally, it may explain Lewy bodies as unused proteins destined for proteolysis (Pollanen et al., 1993; Iwatsubo et al., 1996), and critically, their location in inhibited neurons.

CRFR2-induced serotonin dysregulation may provide a singular point of failure that could explain the many varied signs and symptoms of Parkinson’s. If confirmed, downregulating CRFR2 early in disease progression may reverse the signs and symptoms of Parkinson’s.

### 3.2 Alzheimer’s disease

Alzheimer’s progresses over decades, with increasing memory loss, confusion, cognitive impairment, inattention, disorientation, mood changes, communicative difficulties, autonomic dysfunction and functional impairment (Knopman et al., 2021; Scheltens et al., 2021; Santiago and Potashkin, 2021; Yin et al., 2021; Breijyeh and Karaman, 2020; Tulbã et al., 2020; Zvěřová, 2019, Mukhin et al., 2017). Patient scans show abnormalities in serotonin (Buhot et al., 2000; Geldenhuys, 2011; Claeysen et al., 2015; Kepe et al., 2006), glutamate (Bukke et al., 2020), GABA (Xu et al., 2020), dopamine (Pan et al., 2019), acetylcholine (Davies, 1976; Hampel et al., 2018), histamine (Zlomuzica et al., 2016), norepinephrine (Chen et al., 2022), mitochondria (Wang et al., 2020), and synapses (Tampellini, 2015). Known risks include early life (Lesuis et al., 2018), cumulative (Justice, 2018) and chronic (Futch et al., 2017) stress, antidepressant usage (Wang Y.-C. et al., 2018), insufficient cognitive activity (Vockert et al., 2024), social isolation (Livingston et al., 2024), gender (Beam et al., 2018), certain genes (Van Cauwenberghe et al., 2016), and age. Patient scans/autopsies show protein aggregates (i.e., extracellular amyloid beta plaques and intracellular tau tangles) in the hippocampus, entorhinal cortex, neocortex, amygdala, and basal forebrain. Autopsies also suggest greater decreases in neuronal counts than in normal aging, initially in the hippocampus and entorhinal cortex and spreading to the frontal, temporal and parietal cortices including the dorsal raphé, substantia nigra, nucleus of Mynert and locus coeruleus in the later stages (Mukhin et al., 2017).

Prevailing ideas assume that protein aggregates are neurotoxic and spread in a prion-like manner. Yet, there is no direct evidence of prion-like spread (Walsh and Selkoe, 2016) and such protein aggregates can be present in healthy persons (Jansen et al., 2015; Bennett et al., 2006), absent in patients (Driscoll and Troncoso, 2011), may follow, not precede synaptic loss (Yin et al., 2021), are not Alzheimer’s-specific (Van Arendonk et al., 2023; Medeiros et al., 2011), and are only toxic under contrived conditions (Larson and Lesné, 2012; Ghag et al., 2018). Not surprisingly, a systematic review of the literature shows that protein aggregates fail the minimum Bradford Hill causation criteria in Alzheimer’s (Espay et al., 2019), suggesting that protein aggregates maybe more of an effect than the cause.

The following studies support the case for CRFR2 upregulation in memory, cognitive and autonomic circuits, leading to serotonin dysregulation and symptoms in Alzheimer’s.

- *CRF induces certain symptoms in rats.* Acute high-level CRF, likely activating CRFR2, dose-dependently impairs working memory (Hupalo and Berridge, 2016), cognition (Hupalo et al., 2019), and attention (Hupalo et al., 2021); whereas low-level CRF, likely activating CRFR1, improves attention (Hupalo et al., 2021; Cole et al., 2016). This connects certain symptoms of Alzheimer’s with CRFR2 upregulation.
- *CRF modulates acetylcholine.* In rats, activating CRFR1/CRFR2 releases/suppresses acetylcholine (Pintér et al., 2021), which is drastically reduced throughout the cortex in patient autopsies (Davies, 1976). Acetylcholine is associated with excitability across neural circuits (Picciotto et al., 2012), e.g., it potentiates striatal dopamine in appetitive behaviors (Lemos et al., 2019), but becomes aversive under intense stress (Lemos et al., 2012), which likely upregulates CRFR2. Increasing acetylcholine, via cholinesterase inhibitors, improves patient cognition (Knight et al., 2018). Note that inhibitory/excitatory serotonin receptors bidirectionally modulate acetylcholine release (Nair and Gudelsky, 2004), memory, impulsivity, aggression, learning, cognition and anxiety (Buhot et al., 2000; Carhart-Harris and Nutt, 2017; Hagena and Manahan-Vaughan, 2017; Stiedl et al., 2015). This emphasizes the involvement of CRFR1 in releasing acetylcholine, which CRFR2 upregulation would inhibit.
- *Stress phosphorylates tau protein.* Phosphorylation is critical to the normal functioning of tau protein, involved in axonal transport, synaptic structure and signaling (Tapia-Rojas et al., 2019). In mice, acute stress (likely activating CRFR1) and repetitive stress (likely activating both CRFR1 and upregulated CRFR2) increase phosphorylated tau in the hippocampus at 20 minutes post-stress; however while tau normalizes 24 hours after acute stress, it does not after repetive stress (Rissman et al., 2012). Given that repetitive stress impairs memory (Schwabe, 2025), these data are consistent with inhibited signaling causing phosphorylated tau to remain unused and accumulate under repetitive stress. This suggests that CRFR2 upregulation may induce tau tangles.
- *Autopsies* show decreases in CRF in the frontal, temporal and occipital cortices (Whitehouse et al., 1987), with reciprocal increases in the CRF receptors, correlating with reduced choline acetyltransferase (acetylcholine-synthesizing enzyme) (De Souza et al., 1986). Autopsies also show reductions in 5HT_2A_ in the temporal cortex, correlating with the rate of cognitive decline in patients (Lai et al., 2005). This supports the involvement of the CRF system in Alzheimer’s.

Circuit-specific CRFR2 upregulation and serotonin dysregulation may explain many of the observed abnormalities, including: autonomic symptoms (§2.7); serotonin-related changes in mood, memory, cognition and inattention (Berger et al., 2009), which overlap with the symptoms of serotonin syndrome (Scotton et al., 2019); slow progression, stress-induced symptom-flaring, the influence of early life/cumulative stress, antidepressant usage and gender (§2.7).

Serotonin dysregulation may explain disruptions in other neurotransmitters/neuromodulators, potentially exacerbated by insufficient cognitive activity and social isolation, i.e., disused circuits (Vockert et al., 2024; Livingston et al., 2024). Such inhibition may explain aggregates of unused signaling proteins (amyloid beta, tau) and neuronal atrophy, then evident, *not* in neuronal count (Regeur et al., 1994; Swaab et al., 1994), but in synaptic loss (Davies et al., 1987; DeKosky and Scheff, 1990) that correlates strongly with cognitive impairment (DeKosky and Scheff, 1990; Terry et al., 1991) and only weakly with protein aggregates (Terry et al., 1991; Andrade-Moraes et al., 2013). Disease progression would follow functional networks (Pievani et al., 2011), correlating with tau deposition (Frisoni et al., 2010) and consistent with CRFR2-induced inhibition. The notion of atrophied, not degenerated, neurons may permit their reactivation (Swaab, 2003), which may explain paradoxical lucidity (i.e., temporary return of clarity and communicative abilities) (Mashour et al., 2019; Batthyány and Greyson, 2021; Peterson et al., 2022; Karlawish et al., 2023) as some event, possibly impending death, transmits signals in neurons still capable of signaling.

CRFR2-induced serotonin dysregulation may provide a singular point of failure that could explain many of the signs and symptoms of Alzheimer’s. If confirmed, downregulating CRFR2 early in disease progression may reverse the signs and symptoms of the disease.

### 3.3 Chronic kidney disease

The cause of chronic kidney disease (excluding congenital forms and polycystic kidney disease) is unknown. It is defined by functional loss (i.e., glomerular filtration rate below 60 mL/minute/1.73 m^2^, or albuminuria above 30 mg/24-hours) persisting for 3 months (Chen et al., 2019; Kalantar-Zadeh et al., 2021). Early in the disease, autonomic dysfunction (Grassi et al., 2011) leads to symptoms related to urine production, electrolytes, fluid volume, glucose homeostasis, digestion, breathing and fatigue (Romagnani et al., 2017), later developing cognitive impairment, anxiety, depression (Arnold et al., 2016), movement disorders (Safarpour et al., 2021) and elevated plasma metabolites or uremia (Glassock, 2008). Risk factors include early life (Su et al., 2021; Luyckx and Chevalier, 2022) or cumulative (Bruce et al., 2015) stress, gender (Carrero et al., 2018), hyperglycemia, hypertension, cardiovascular disease and autoimmunity (Romagnani et al., 2017; Fogo, 2007; Webster et al., 2017). Chronic kidney disease is generally attributed to nephron injury/loss, but this is difficult to separate from age-related atrophy (Denic et al., 2017; Hughson et al., 2020), and inconsistent with the fact that kidney function, can improve at any stage (Weis et al., 2013; Liu et al., 2021). Non-kidney symptoms are generally assigned to uremic toxins, but these are highly variable, not necessarily toxic (Duranton et al., 2012; Lim et al., 2021) and fail to explain selectivity for only certain symptoms.

The case for CRFR2 upregulation in autonomic circuits causing chronic kidney disease is supported in rats, where intravenous CRF (Gutkowska et al., 2000), at CRFR2-activating doses (Hupalo and Berridge, 2016; Hupalo et al., 2019, 2021), or subcutaneous CT38, induce dose-dependent reductions in glomerular filtration rate, urine volume and electrolytes (Figures 3G, H), likely via increased norepinephrine (Kopp, 2018; Figure 3K).

More broadly, circuit-specific CRFR2 upregulation and serotonin dysregulation may explain many of the observed abnormalities, including: autonomic (§2.7), psychological and movement symptoms (Berger et al., 2009; Scotton et al., 2019), slow progression, stress-induced symptom-flaring and the influence of early life/cumulative stress and gender (§2.7). CRFR2-induced serotonin increase may explain the slowed progression and reduced mortality that occurs with cholinesterase inhibitors (Xu et al., 2023), which offset CRFR2 suppression of acetylcholine; and with *high-dose* selective serotonin reuptake inhibitors (Dev et al., 2014), which induce a compensatory decrease in brain serotonin (Andrews et al., 2015), so greater decrease in serotonin at high-dose than low-dose. It may also explain inhibitory (Chamienia and Johns, 1994)/excitatory (Kaur and Krishan, 2020) serotonin receptors bidirectionally affecting autonomic activity and releasing norepinephrine to affect renal blood flow, tubular absorption and renin production in the kidneys (Kopp, 2018). Finally, a circuit explanation allows for a change in signaling (e.g., serotonin decreasing, CRFR2 downregulating, etc.) to *improve* kidney function, at any stage.

CRFR2-induced serotonin dysregulation may also explain hyperglycemia (associated with type 2 diabetes), hypertension and cardiovascular disease—each risk factors for chronic kidney disease.

- *CRFR2 upregulation in the ventromedial nucleus of the hypothalamus may explain hyperglycemia.* Studies show that the CRF system influences glucose via the pancreas, liver and skeletal muscle. Acute CRF modulates pancreatic hormones via the vagus nerve (Payne et al., 2020), e.g., in hypoglycemic rats, activating CRFR2, suppresses glucagon and releases insulin (McCrimmon, 2006), while activating CRFR1 has the opposite effects (Cheng et al., 2007), i.e., the incorrect and correct counter-regulatory response, respectively. Acute CRFR2 stimulation, acts via the vagus nerve (Cardin et al., 2002), to modulate hepatic glucose release in stressed mice, both short-term (glycogenolysis) and long-term (gluconeogenesis) (Liu et al., 2024). Acute CRFR2 stimulation also modulates glucose uptake in skeletal muscle, e.g., a single intraperitoneal dose of urocortin2 in mice, reduces muscle uptake, inducing sustained (2-hour) insulin resistance (Flaherty et al., 2023), an effect mediated by the mechanistic target of rapamycin or mTOR (Chao et al., 2015). Chronic CRFR2 activation, via genetic excess of urocortin2, desensitizes CRFR2 (likely via downregulation) and amplifies insulin sensitivity (Flaherty et al., 2023). Similarly, genetic excess of urocortin3 (Jamieson et al., 2011) or continuous infusion of CT38 (Figure 3J), likely downregulating CRFR2, prevents diet-induced obesity and results in a lean body composition. Note that recent studies assign type 2 diabetes to nutritional excess (Taylor, 2024; Klein et al., 2022), which is consistent with upregulated CRFR2 as an adaptation that can be reversed by downregulation, behaviorally or pharmacologically. Note further that serotonin is involved in all aspects of glycemic control, with inhibitory and excitatory receptors mediating bidirectional effects (Serlie, 2020; Cai et al., 2022; Babic and Travagli, 2016; Gehlert and Shaw, 2014; Berglund et al., 2013; Gilles et al., 2005; Voigt et al., 2004; Darvesh and Gudelsky, 2003), via norepinephrine (Ahrén et al., 1987; Dunning et al., 1988; Luo et al., 1999) and the autonomic system (Hyun and Sohn, 2022). Thus, CRFR2 in the ventromedial hypothalamus could set central glucose, and upregulation could disrupt insulin, glucagon, hepatic glucose release and peripheral glucose uptake, promoting hyperglycemia.
- *CRFR2 upregulation in the paraventricular nucleus of the hypothalamus may explain hypertension.* Studies show that optogenetic activation of CRFR2 in the hypothalamus (Wang et al., 2019), or acute intracerebroventricular CRF (Fisher et al., 1982), elevate mean arterial pressure, likely via the nucleus of the solitary tract (Wang L. A. et al., 2018). Thus, CRFR2 in the hypothalamic neurons projecting to the nucleus of the solitary tract could set central homeostatic blood pressure, and upregulation could induce hypertension, which is associated with an overactive renin-angiotensin-aldosterone system (Ferrari, 2013), producing nitric oxide within the podocytes via intracellular calcium, whose excess is implicated in podocyte foot process effacement, detachment and proteinuria (Semenikhina et al., 2024). Note that inhibitory/excitatory serotonin receptors affect blood pressure bidirectionally (Lin and Lin, 1996). Note further that non-targeted CRFR2 activation, e.g., high-dose intravenous CRF in rats (Hermus et al., 1987) or subcutaneous CT38 in humans (Figure 3C), *reduces* diastolic blood pressure, but this effect likely involves peripheral CRFR2 in the vasculature (Wiley and Davenport, 2004; Takefuji and Murohara, 2019).
- *CRFR2 upregulation in the posterior nucleus of the hypothalamus may explain elevated heart rate.* Studies show that activating CRFR1 and CRFR2 additively, by direct injection into the posterior hypothalamic nucleus, elevates rat heart rate, via the cardiac sympathetic nerve, not the vagus nerve, without affecting arterial pressure (Gao et al., 2016). Thus, CRFR2 in the posterior hypothalamic nucleus could set resting heart rate, and upregulation could elevate heart rate, which is associated with increased all-cause mortality in chronic kidney disease (Saito et al., 2024). Note that inhibitory/excitatory serotonin receptors affect heart rate bidirectionally (Chamienia and Johns, 1994; Neumann et al., 2023).

Hyperglycemia, hypertension, cardiovascular disease and certain autoimmunities (e.g., immunoglobulin A nephropathy, complement 3 glomerulopathy) are seen as risks for chronic kidney disease. It is possible that these conditions are *not* risks *per se*, but rather reflect a common origin, namely CRFR2 upregulation in the hypothalamus/brain stem.

CRFR2-induced serotonin dysregulation may provide a singular point of failure that could explain the symptoms of chronic kidney disease, hyperglycemia, hypertension and elevated heart rate. If confirmed, downregulating CRFR2 early in disease progression may reverse the signs and symptoms of these diseases.

### 3.4 CRF-serotonin in systemic regulation

The foregoing shows that CRF-serotonin could regulate individual and autonomic functions. It could also regulate the endocrine and immune systems, and play a role in certain epigenetic modifications, all ultimately influencing the aging process.

- *CRF-serotonin and the endocrine system.* The hypothalamus controls the pituitary, which releases CRF to increase adrenal and pancreatic activity, while reducing thyroid, gonadal and pineal activity (Dedic et al., 2018; Inda et al., 2017; Gore, 2013; Hiller-Sturmhöfel and Bartke, 1998; Kellner et al., 1997). Thus, CRFR2 upregulation could affect basal cortisol, and promote hyperglycemia (Payne et al., 2020; McCrimmon, 2006; Cheng et al., 2007), hypothyroidism (Castillo-Campos et al., 2021) and hypogonadism (Kageyama, 2013; Raftogianni et al., 2018).
- *CRF-serotonin and the immune system.* Recent rat studies show that the immune system is controlled by the nucleus of the solitary tract, which receives pro-/anti-inflammatory signals via the vagus nerve (Jin et al., 2024; Tränkner et al., 2014). CRF-serotonin modulates the nucleus of the solitary tract (Sévoz-Couche and Brouillard, 2017). Not surprisingly, CRF-serotonin is implicated in autoimmunity, e.g., multiple sclerosis (Huitinga et al., 2003; Purba et al., 1995; Wan et al., 2020), inflammatory bowel disease (Buckinx et al., 2011; Paschos et al., 2009; Wan et al., 2020) and rheumatoid arthritis (Chikanza et al., 1992; Eijsbouts and Murphy, 1999; McEvoy et al., 2001; Wan et al., 2020). CRF-serotonin is also implicated in sepsis (Gonzalez-Rey et al., 2006; Mota et al., 2017) and cancer (Androulidaki et al., 2009; Arranz et al., 2010; Argilés et al., 2008; Hao et al., 2008; Wang et al., 2009; Jin et al., 2019; Balakrishna et al., 2021), which is increasingly connected with the nervous system (Venkatesh et al., 2015; Venkatesh et al., 2019; Venkataramani et al., 2019; Krishna et al., 2023; Taylor et al., 2023; Aabedi et al., 2021). These studies show CRF-serotonin dysregulation in these conditions, and, accounting for the CRF dose-response, they suggest that CRFR2 maintains the immune response. By implication, CRFR2 upregulation may play a role in autoimmunity, while stimulating CRFR2 may be beneficial in sepsis and cancer.
- *CRF-serotonin and epigenetics.* During early development, and particularly under early life stress, epigenetic change (DNA methylation, histone acetylation, etc.) has been shown in specific neuronal genes regulating the stress response, monoamines and neuropeptides, including 5HT_1A_ and CRF (Rahman and McGowan, 2022). Histone acetylation has been implicated in age-dependent memory impairment (Peleg et al., 2010), but also in enhancing memory and synaptic plasticity in hippocampal neurons, where it is contingent upon on second messenger effects (Vecsey et al., 2007). This latter point is critical. It suggests that epigenetic change is driven by neuronal/cellular activity, or inactivity. That is, if CRFR2 upregulates in a neuron, it likely induces CRFR2-related epigenetic modifications in *that* neuron, with the resulting alterations in downstream signaling inducing further epigenetic modifications in downstream neurons and systems. In this way, CRFR2-serotonin signaling changes potentially induce neuron-specific epigenetic alterations in the nervous (Sweatt, 2009; Borodinova and Balaban, 2020; Qureshi and Mehler, 2018), autonomic (Dos Santos Oliveira et al., 2023; Wiegand et al., 2021; Wise and Charchar, 2016; Bayles et al., 2012), endocrine (Plunk and Richards, 2020; Zhang and Ho, 2011) and immune (Obata et al., 2015; Jasiulionis, 2018) systems. Note that given the apparent long duration of effect noted in the proof-of-concept trial (§2.8), it is possible that CRFR2 downregulation could reverse prior epigenetic changes.
- *CRF-serotonin in aging.* The aging process manifests as a gradual decline in function and an increased susceptibility to certain diseases, influenced by 12 hallmarks (López-Otín et al., 2023; Gorgoulis et al., 2019). The theory presented here views these hallmarks (italicized below) as direct/indirect effects of cumulative CRFR2 upregulation over the lifespan (McEwen, 2017), which, in an age-dependent manner, could dysregulate brain signals, *altering intercellular communication*. Dysregulated signaling would impair function and increase the incidence of ACD (GBD 2019 Acute and Chronic Care Collaborators, 2025; Buttorff et al., 2017) and the dysfunction of the autonomic (Alrosan et al., 2024), endocrine (Van Den Beld et al., 2018; Xing et al., 2023) and immune (Goyani et al., 2024; Yu et al., 2024) systems. In turn, autonomic dysfunction could inhibit gastrointestinal motility leading to *dysbiosis* (Akbarali and Dewey, 2019); endocrine dysfunction in the hypothalamus could activate the mechanistic target of rapamycin to *dysregulate nutrient sensing* (Hu et al., 2016); and immune dysfunction could lead to *chronic inflammation* and *stem cell exhaustion*. Dysregulated signaling also affects cellular activity so could induce *cellular senescence* (Gorgoulis et al., 2019), via *epigenetic alteration* and *telomere attrition* (unlikely in neurons); *mitochondrial dysfunction* via CRF activating nuclear factor-κB transcription (Battaglia et al., 2020); *loss of proteostasis* compounded by *disabled macroautophagy* via CRFR1/CRFR2 activating the mechanistic target of rapamycin (Jin et al., 2019); and *genomic instability* (unrelated to signaling *per se*, but induced by drugs, radiation, etc.). Note that much of this dysregulated signaling involves the hypothalamus, where activating/inhibiting nuclear factor-κB transcription, accelerates/decelerates aging in mice (Zhang et al., 2013). Note further that sarcopenia, the age-related decline in muscle mass and composition (von Haehling et al., 2010), is reversed by chronic high-level CRFR2 stimulation (Hinkle et al., 2011), which may downregulate CRFR2 (Figure 3L).
- CRFR1/CRFR2-serotonin are implicated in autism (Tsilioni et al., 2014; Martinon and Dabrowska, 2018; Jørgensen et al., 2003; Cataldo et al., 2018), epilepsy (Tiwari et al., 2022; Bagdy et al., 2007), post-traumatic stress disorder (Elharrar et al., 2013; Sullivan et al., 2013), chronic pain (Ji and Neugebauer, 2007; Kim et al., 2014), addiction (Lowery et al., 2010; Müller and Homberg, 2015), attention-deficit-hyperactivity-disorder (Hupalo et al., 2021; Cole et al., 2016; Banerjee and Nandagopal, 2015), depression (Gold, 2015; Jauhar et al., 2023), anxiety (Todorovic et al., 2007; Gordon and Hen, 2004), sleep (Ono et al., 2020; Nakamaru-Ogiso et al., 2012), migraine (Sarchielli et al., 2008; Deen et al., 2018), Huntington’s disease (De Souza et al., 1987; Cross et al., 1986), amyotrophic lateral sclerosis (Klimek et al., 1986; Yang et al., 2023), hypothyroidism (Okada et al., 2007; Bauer et al., 2002), chronic dyspnea (Pereira et al., 2021; Mann et al., 2009; Hilaire et al., 2010), central fatigue (Pereira et al., 2021; Cotel et al., 2013; Perrier and Cotel, 2015), irritable bowel syndrome (Porcher, 2005; Stasi et al., 2014), interstitial cystitis (Jhang et al., 2019; Yoshimura et al., 2014; Chen et al., 2024), endometriosis (Vergetaki et al., 2013; Tian et al., 2024), metabolic dysfunction-associated steatotic liver disease (Parisse et al., 2025; Pagire et al., 2024), and alopecia (Katsarou-Katsari et al., 2001; Pejcic and Paudel, 2022). More broadly, the CRF system is implicated in maintaining muscle mass (Argilés et al., 2008; Hinkle et al., 2011; Hinkle et al., 2003a; Hinkle et al., 2003b; Hinkle et al., 2004; Hall et al., 2007; Hinkle et al., 2007), obesity (Lu et al., 2015; Borg et al., 2019), motor learning (Takeuchi et al., 2024), sensory perception (Liu et al., 2022; Graham et al., 2011; Shin et al., 2023; Heinrichs et al., 1991; Harrison and McLoon, 2006), acute pain (Zheng et al., 2020), pruritus (Wang et al., 2022), hepatic function (Paschos et al., 2013), motivation (Welberg, 2012), bipolar disorder (Guo et al., 2022), and social behavior (Hostetler and Ryabinin, 2013).

## 4 Discussion

This theory of brain signaling proposes that CRF acts via CRFR1 and CRFR2 to modulate serotonin in functionally-dedicated circuits. In turn, serotonin activates inhibitory and excitatory receptors on effector neurons, to architect precise, real-time, situationally-specific signals that elicit other inputs to regulate normal function, both involuntary (e.g., homeostatic, autonomic, endocrine, immune, sensory) and voluntary (e.g., motor, cognitive, emotional). Circuit-specific CRFR2 can become permanently upregulated, thereby dysregulating the signals to cause chronic symptoms and ACD.

It is well established that: (i) CRF is released in situations requiring adaption to meet internal and external conditions; (ii) CRF acts via CRFR1 and CRFR2 to modulate serotonin release from the raphé; (iii) CRFR2 can permanently upregulate in the raphé; (iv) the CRF and serotonin systems overlap throughout the brain; and (v) serotonin stimulates co-expressed inhibitory and excitatory receptors on individual neurons. The only novel conjecture of this theory, was extending known CRF-serotonin interaction in the raphé, to the dedicated circuits. This is supported by hundreds of independent studies showing that CRF-serotonin *bidirectionally* regulates other neurotransmitters/neuromodulators and numerous functions (e.g., thermoregulation, movement, memory, glomerular filtration rate); yet *unidirectionally* dysregulates, consistent with CRFR2 upregulation, to cause individual chronic symptoms (e.g., low temperature, impaired movement, memory loss, reduced glomerular filtration rate). This broad data set obviates the problem of individual study replicability (Jarvis and Williams, 2016), and the consistent pattern across species, model systems and experimental methods, arguably provides a more reliable foundation for understanding physiological processes than any single study taken in isolation (Ineichen et al., 2024; Munafò and George, 2018; Lawlor et al., 2017; Sena et al., 2014; De Vries et al., 2014; Russo and Williamson, 2007). The issue of possible citation bias is abrogated by the failure to find studies that could not be accommodated by the theory, and that, until now, there would be no reason to test CRF-serotonin control of function. While much work remains to be done to map the dedicated circuits and their inputs and cross-talk, a situationally-specific, CRF-serotonin trigger signal provides an intriguing foundation.

Box 1 **Interpretation in Parkinson’s.**

Parkinson’s autopsies suggest reduced dopamine neuron counts in the substantia nigra (decades after symptoms onset), and intracellular Lewy bodies that aggregate various proteins, e.g., alpha-synuclein (involved in neurotransmission), ubiquitin, neurofilaments, etc.

Reduced dopamine neuron counts at autopsy rely on staining for tyrosine hydroxylase or neuromelanin pigment. Yet, these proteins, and in fact most counting methods, are influenced by neuronal activity, so a low reading could indicate neuronal inhibition, and not neuronal loss.

Lewy bodies are thought to form because alpha-synuclein misfolds. Yet there is no direct evidence of misfolding causing aggregation, and the presence of other proteins suggests that Lewy bodies might be unused proteins destined for recycling, explaining their presence in healthy persons.

Lewy bodies are thought to be toxic. Yet there is no direct evidence of toxicity, which is assumed from abnormal scans of receptors, transporters, mitochondria, synapses, microglia, etc, and ostensibly reduced neuron counts at autopsy, when these could be adaptations to neuronal inhibition, with prolonged signal loss potentially leading to neuronal death.

The selectivity for the nigra is thought to result from its high level of activity and large neurons. Yet this does not explain Lewy bodies in healthy persons selecting other brain regions.

Progression from the nigra is thought to involve misfolded alpha-synuclein spreading in a prion-like fashion into adjacent neurons. Yet there is no direct evidence for prion-like spread, and the development of downstream Lewy bodies might result from downstream inhibition.

In contrast, prevailing ideas do not address the normal regulation of function *per se*, so cannot assign abnormalities/chronic symptoms to departures from normal regulation. Instead, they are assumed to result from cellular damage, but it is important to understand the underlying data. Brain measurement involves indirect proxies from which abnormalities are interpreted, but this assumes static neurons/cells that can be compared, when in fact, they adjust to signaling demands. Box 1 presents Parkinson’s observations and their interpretations under prevailing thinking, highlighting the interpretations for which there is no direct evidence (i.e., neuronal loss, Lewy body formation due to alpha-synuclein misfolding, Lewy body toxicity, selectivity for the nigra, disease progression). Perhaps most glaring, is the failure to acknowledge the possibility of aberrant neuronal signaling, despite this being the fundamental activity of the neurons, and in which, alpha-synuclein participates. The theory presented here, essentially dysregulated signaling rather than cellular damage, may explain the many abnormalities of ACD and help to unify the research.

If confirmed, this theory has important consequences:

- *Any circuit-engaging stimuli, if intense or repetitive enough, can upregulate CRFR2 in the appropriate circuit(s), to cause chronic symptom(s)*. Such stimuli include microbes, toxins, physical/mental trauma, and, less obviously, elevated glucose, substance abuse, muscle disuse, increased reading/decreased outdoor time, etc., potentially leading to insulin resistance/hyperglycemia, tolerance/addiction, atrophy and myopia (Li et al., 2025), respectively. Medications might be stimuli, e.g., hypertensive and arrhythmic drugs can induce lupus (Vedove et al., 2009; Timlin et al., 2019), tuberculosis drugs can impair vision (Aggarwal et al., 2021), fluoroquinolone antibiotics can weaken tendons and cause arrhythmic, glycemic, gastrointestinal and psychiatric symptoms (Baggio and R Ananda-Rajah, 2021), antihistamines (Adler and Baumgart, 2020) and benzodiazepines (Stewart, 2005) can impair cognition, proton pump inhibitors can decrease bone density (Smaoui et al., 2024)—all these side effects link to serotonin. Finally, vaccines might also be stimuli, e.g., in mice, 3 subcutaneous injections of heat-killed bacteria (*no* adjuvant), intended to simulate microbial exposure in the wild, induced persistent (4 weeks post-injection) behavioral changes, and increased tryptophan hydroxylase 2 (the rate-limiting enzyme for serotonin synthesis) in the dorsal raphé (Reber et al., 2016). These findings are consistent with this theory, and though preliminary, raise concerns. The vaccine debate relies on long-term epidemiological statistics, which are affected by changes in sanitation, nutrition, environmental toxins, disease diagnostic criteria, medical practice, maternal care, daily stress, herd immunity, lack of vaccine-naive controls, etc, all open to interpretation. This theory provides the means to assess vaccine safety mechanistically (via serotonin changes in the dorsal raphé of mice), and potentially, the ability to reverse side effects in patients.
- *The translation of circuit-engaging stimuli into serotonin signals means that different stimuli can be additive, and their effects can be cumulative*. Thus, CRFR2 upregulation, even in an individual circuit, could have more than one cause, making causality difficult to determine, e.g., microbial absence does not rule out microbial causality, but equally, microbial presence does not, of itself, imply microbial causality. Cumulative effect ties the dysregulation of a circuit to its history, which explains why the risk of ACD increases with cumulative exposure to infections (Bjerke, 2011), toxins (You et al., 2024; Todd et al., 1996) or trauma (Stewart et al., 2021; Mock and Arai, 2011), and why the impact of poly-pharmacy is unpredictable (Doumat et al., 2023; Wastesson et al., 2018).
- *Animal models based on attempts to replicate symptoms become questionable*, as they do not explicitly upregulate CRFR2, although an intervention might do so. Note that, if the CRF system plays a fundamental role in body regulation and adaptation, CRF system-related knock-outs/ins could change the natural response of the system to provocation, and thus the use of such models has its limitations.
- *Biomarkers become indirect*, as neither CRFR2 upregulation nor net serotonin signals can be measured at the neuronal level, and anything measured downstream (e.g., bodily fluids, biopsies) will typically reflect multiple influences.

Despite these issues, this theory is easily tested to a binary outcome. That is, if CRFR2 plays a causal role in ACD, a bolus of a CRFR2 agonist in animals, either directly into a specific brain region or systemically to mimic specific/general provocations, should induce dose-dependent signs indicative of ACD symptoms (Figure 3), and CRFR1 and CRFR2 agonist doses above their no-observed-effect-levels should modulate a given function bidirectionally. Alternatively, a bolus of a CRFR2 agonist at a concentration *slightly above* its no-observed-effect-level, or for that matter day-to-day stress, should transiently worsen patient symptoms (Figure 5A), so could aid in diagnosis. Finally, a therapeutic infusion of an agonist, at a concentration maintained *below* its no-observed-effect-level should permanently improve patient symptoms (Figures 5B, C). Such a trial would be expected to be safe, and could even be delivered as a number of short infusions for added safety. It should have a rapid onset of effect (from disinhibiting neuronal signals), permitting short trials (days), especially if objective tests exist (e.g., 6-min walk test in Parkinson’s, glomerular filtration rate in chronic kidney disease).

In conclusion, the authors have proposed a specific basis for brain signals to regulate normal function and become dysregulated in ACD, with the possibility that normal regulation may be restored to reverse the signs and symptoms of ACD.
